# The electrocardiographic profile of patients with angina pectoris

**Published:** 2009

**Authors:** Carmen Ginghina, Catalina Ungureanu, Aurora Vladaia, B.A. Popescu, Ruxandra Jurcut

**Affiliations:** * “Prof. Dr. C.C. Iliescu” Institute of Cardiovascular Diseases, Bucharest

## Abstract

Angina pectoris is a common disabling disorder and a clinical syndrome, caused by myocardial ischemia; an imbalance between myocardial oxygen supply and myocardial oxygen consumption. Thus, ischemia produces a typical series of events such as metabolic and biochemical alterations which lead to impaired ventricular relaxation and diastolic dysfunction, impaired systolic function, and electrocardiographic abnormalities and painful symptoms of angina. Transmembrane ionic currents are responsible for the cardiac potentials that are recorded as the electrocardiogram (ECG).

The electrocardiographic profile of patients with angina pectoris is variate. The electrocardiogram provides critical information for both diagnosis and prognosis, particularly when a tracing is obtained during the episodes of pain. A completely normal electrocardiogram does not exclude the possibility of acute coronary syndrome. Serial ECG tracings improve the clinician’s ability to diagnose acute and chronic coronary syndromes. The ECG may assist in clarifying the differential diagnosis if taken in the presence of pain. The resting ECG also has an important role in risk stratification.

Exercise ECG is more sensitive and specific than the resting ECG as far as myocardial ischemia detection is concerned, and it represents the test of choice which helps identifying inducible ischemia in the majority of patients suspected of stable angina.

Angina pectoris, commonly known as angina, is a severe chest pain produced by the ischemia of the heart muscle, generally resulting into obstruction or spasm of the coronary artery (the heart's blood vessels). Coronary artery disease, the main cause of angina, appears due to atherosclerosis of the cardiac arteries. The term derives from the Greek *ankhon* ("strangling") and the Latin *pectus* ("chest"), and can therefore be translated as "a strangling feeling in the chest".

The heart, an aerobic organ, relies almost exclusively on the oxidation of substrates for energy generation. The coronary circulation supplies the heart with oxygen and nutrients in order to maintain cardiac function and thus supply the remainder of the body with blood. Imbalance in myocardial oxygen demand and supply can produce myocardial ischemia with contractile cardiac dysfunction, arrhythmias, infarction, and possibly death [**1**].

Ischemia presents complex time-dependent effects on the electrical properties of myocardial cells. Severe, acute ischemia can reduce the resting membrane potential, shorten the duration of the action potential in the ischemic area, and decrease the rise rate and the amplitude of phase *0*. These changes cause a voltage gradient between normal and ischemic zones, that leads to current flow in these regions. The currents of injury are represented by deviation of the ST segment on the surface ECG. All patients suspected of angina pectoris based on symptoms, should have a 12-lead ECG resting recorded. It should be emphasized that a normal resting ECG is not uncommon even in patients with severe angina and does not exclude the diagnosis of ischemia. About 1 - 6 % of the patients with acute chest pain are subsequently diagnosed with acute myocardial infarction, although the prognosis for patients with a normal or near - normal ECG is higher than that of patients with clearly abnormal ECGs at admission [**2**].

The ECG remains a key test in the diagnosis of acute and chronic coronary syndromes. The findings varies considerably, depending on four major factors: the duration of the ischemic process (acute vs. chronic); its extent (transmural vs. nontransmural); its topography (anterior vs. inferior-posterior and right ventricular) and the presence of other underlying abnormalities (left bundle branch block - LBBB, Wolff-Parkinson-White syndrome, or pacemaker patterns) that can mask or alter the classic patterns.

The ECG may clarify the differential diagnosis allowing detection of dynamic ST-segment changes in the presence of ischemia or by identifying features of pericardial disease. An ECG during pain may be particularly useful if vasospasm is suspected.

Such information may be helpful in defining the mechanisms responsible for chest pain, in selecting an appropriate further investigation, or in tailoring individual patient treatment. The resting ECG has an important role in risk stratification; risk refers primarily to the fear of cardiovascular death, but the term is often said to incorporate cardiovascular death and myocardial infarction, or in some cases even wider combinations of cardiovascular end-points.

It is not common to equate the severity of angina with the risk of fatal cardiac events. There is a weak relationship between the severity of pain and the degree of oxygen deprivation in the heart muscle (i.e. there can be severe pain with little or no risk of a heart attack, and a heart attack can occur without pain).

Exercise ECG is more sensitive and specific than the resting ECG in detecting myocardial ischemia. There are numerous reports and meta-analyses of the performance of exercise ECG for the diagnosis of coronary disease [**3**]. Using exercise ST depression less than 0.1mV or 1mm to define a positive test, the reported sensitivity and specificity for the detection of significant coronary disease range between 23-100% (mean 68%) and 17-100% (mean 77%), respectively. ECG changes associated with myocardial ischemia include horizontal or down-sloping ST-segment depression or elevation [≥ 1mm (0,1mV) for ≥ 60-80ms after the end of the QRS complex], especially when these changes are accompanied by chest pain suggestive of angina, they occur at a low workload during the early stages of exercise and persist for more than 3 minutes after exercise. Increasing the threshold of a positive test to ≥ 2mm (0.2mV) ST-depression, will increase specificity at the expense of sensitivity.

Exercise ECG testing does not present a diagnostic value in the presence of LBBB, paced rhythm, and Wolff-Parkinson-White syndrome, in which cases, the ECG changes cannot be evaluated. In addition, false-positive results are more frequent in patients with abnormal resting ECG in the presence of left ventricular hypertrophy, electrolyte imbalance, intraventricular conduction abnormalities, and use of digitalis. Exercise ECG testing is also less sensitive and specific to women. The ECG tracing is also an important indicator for the evolution of coronary heart disease.

So, we present some clinical cases with particular ECG tracing and their echocardiographic and angiographic features, by using a didactic approach.

## ST – segment depression

 The earliest electrocardiographic change often associated with ischemia is ST-segment depression.

In 2002, the European Heart Journal presented the results of a retrospective analysis which included 2457 patients with unstable coronary artery disease. The conclusion was: ST-segment depression is associated with an 100% increase in the occurrence of three-vessel/left main disease and to an increased risk of subsequent cardiac events. In these cases, patients presented an early invasive strategy which substantially decreased death/myocardial infarction [**5**].

The underlying study was published in 1993 by the Cardiovascular Research Institute in Maastricht, Netherlands. The aim of the study was to assess the value of the electrocardiogram recorded during chest pain in order to identify high-risk patients with 3-vessel or left main stem coronary artery disease. Electrocardiograms recorded during chest pain were compared with the ones from a symptom-free episode. Left main and 3-vessel coronary artery disease showed a frequent combination of leads with abnormal ST segments: ST-segment depression in leads I, II and V4-V6, and ST-segment elevation in lead aVR. The negative predictive and positive accuracy of this pattern were 78 and 62%, respectively [**6**] 

**Case I:**
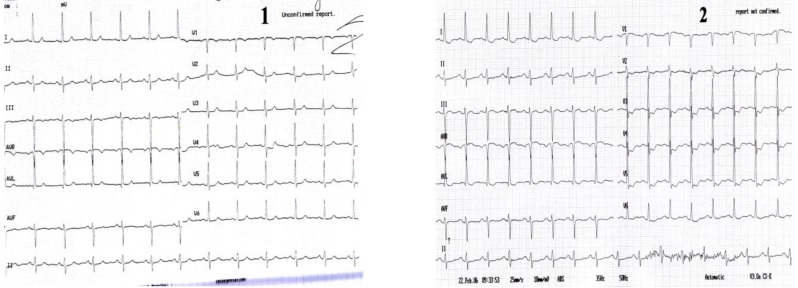
*Electrocardiograms of a 68 year-old woman, with prior anterior myocardial infarction and worsening of angina symptoms which became more frequent and also occurred at rest*. **Resting ECG** (1) shows a sinus rhythm of 72 beats/min, with negative T wave in leads III and aVF. **During anginal pain** (2) note: ST-segment depression in leads I, aVL and V2-V6 and ST-segment elevation in lead III, aVR and V1.

**Figure:**
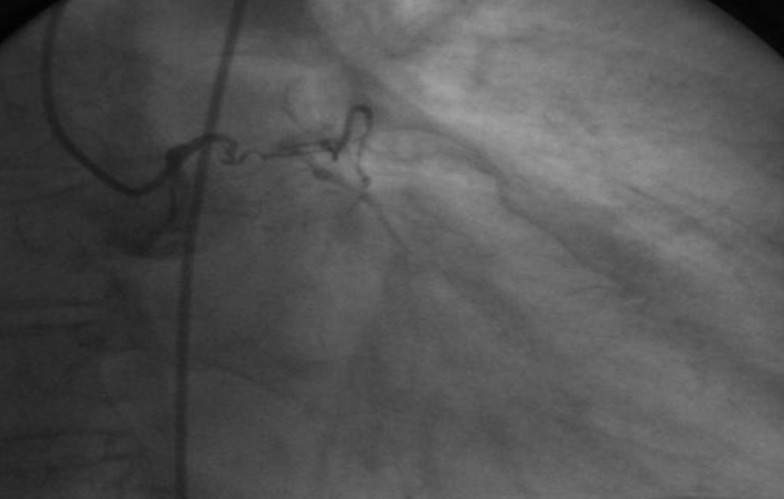
**Coronary arteriography revealed**: left main chronic occlusion and a higher number of mildly stenotic and non-stenotic plaques in the right coronary artery with rich collaterally branches.

**Case II:**
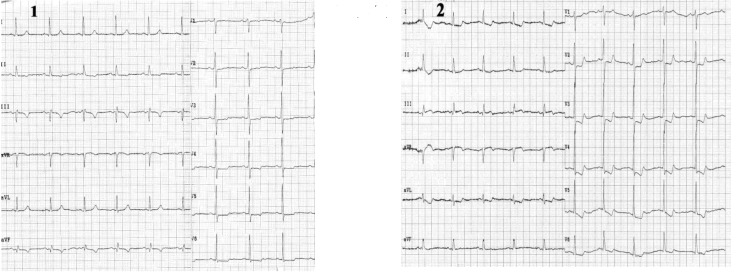
*The electrocardiograms of a 62 year-old woman, corpulent, with concomitant disorders such as dyslipidemia and hypertension. She was admitted with night episodes of typical angina, with recent onset within 2 months*. **Resting ECG** (1) shows us: a regular sinus rhythm of 70 beats/min, Q wave in leads III, aVF, negative T wave in leads III, aVF, and diphasic T wave in leads II, V2-V6, 0.5 mm ST-segment depression in leads V3-V6. **During anginal pain** (2) we note: increased ST - segment depression in leads V3-V6 (to 2mm) and additionally to resting ECG features, ST - segment depression in leads III and aVF. **Coronary arteriography:** (three vessel coronary artery disease (stenosis in the left anterior descending coronary artery (LAD) II of 90% and in LAD III of 50-60%, stenosis in the proximal intermediar coronary artery of 50% - an important vessel, and occlusion in right coronary III).

## Negative T-wave

Ischemic negative T waves often occur associated with ST –segment depression, becoming normal again after the end of the anginal pain. Frequently, the inversion of T waves continues for some time after the normalization of the ST-segment. In practice it was noticed that there is no relationship between the leads with negative T wave and a certain coronary artery disease.

A study from Tampere University Hospital, Finland, published in 2004, concluded that transient ST-segment depression and a negative T wave present in leads V4-5 during anginal pain, predicts left main, left main equivalent, or severe three-vessel coronary artery disease with high sensitivity and specificity. In patients with ST-segment depression and a positive T wave there is a high probability of one-vessel disease [**7**].

For example, T-wave inversion in the anterior precordial leads takes many forms, has multiple causes, and is a normal variant in the persistent juvenile T-wave pattern. In 1982, de Zwaan et al drew attention to the specificity of a unique type of anterior T-wave inversion for ischemia and/or injury in the distribution of the LAD. The ST segment and the first half of the T-wave are essentially normal. At its peak the T-wave makes a sharp >90° turn, and its terminal portion is negative. This change came to be known as Wellens' warning. It is usually seen hours or days after myocardial ischemic pain subsided. The natural history of Wellens' syndrome is similar to anterior wall acute myocardial infarction. During pain, T-waves are usually upright with ST elevation or ST depression. Depending on the intensity of the ischemia and/or of the injury, the T-waves may return to normal or become deeply, symmetrically inverted, the so-called Pardee T-waves. As the deep inversion resolves over a period of days or months, the pattern of terminal T inversion may be seen again [**8**].The clinician will most probably encounter these changes in the sensation-free patient. Many subsequent studies showed that these patients, who show characteristic ST-T segment changes in the precordial leads on or shortly after admission, have a critical stenosis high in the left anterior descending coronary artery [**9**]. Once the definitive management of the stenosis has been realized, the changes resolve with the normalization of the electrocardiogram. It is vital that the physician recognizes these changes and the association with critical LAD obstruction and significant risk for anterior wall myocardial infarction. The moment these signs are observed, urgent coronary angiography and, when possible, coronary revascularization should be done in patients with unstable angina, who show this ECG pattern [**10**]. Although precordial T-wave inversion has originally been described as presenting proximal LAD lesions, ischemia in the territory of the left circumflex (LCx) and occasionally right coronary artery (RCA) can also cause precordial T-wave inversion.

**Case III:**
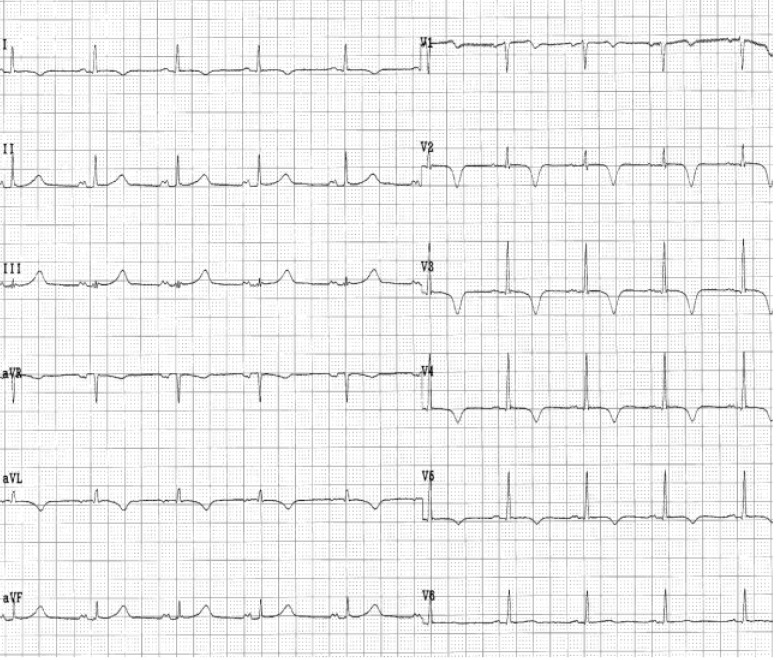
*Electrocardiogram of a 62 year-old man who is admitted with new onset angina, without anterior cardiovascular diseases*. The resting **ECG** shows: a regular sinus rhythm of 60 beats/min, AQRS +30°, negative T-wave in leads I, aVL. **Echocardiographic**: left ventricular antero-apical wall hypokinesia. **Coronarographic**: seriated stenosis in first diagonal branch of 80-90%.

 It is important to know that: T-wave inversion has a wide range of etiologies, from a normal variant to hypertrophic cardiomyopathy, pericarditis, and life-threatening myocardial ischemia. The majority of T-wave inversions fall into a category of "nonspecific ST-T-wave abnormalities" and account for 50% to 70% of abnormal tracings in general hospital populations. The interpretation of these ECGs is primarily based on correlation with available clinical data. There are no established electrocardiographic criteria that adequately distinguish between post-pacing precordial T-wave inversions, known as cardiac memory and mimic anterior myocardial ischemia. It is vital that the physician recognizes these changes in a clinical context in order to make a good diagnosis and to assure an optimal management treatment.

## Positive T-wave

A tall, peaked, positive and symmetric T-wave occurring in the presence of a ST segment elevation and normal T intervals may be the only preliminary electrocardiographic finding in an ischemic heart. On the other hand it may be innocent.

Although it has often been mentioned that the presence of positive, sharp and tall T-waves could be either a sign of subendocardial ischemia or of impending infarction, their occurrence has not received much attention. This particular type of T-wave is seen in the precordial leads, mostly in V2, V3, and V4. In this study, Freundlich found out that about 58 % of all patients with peaked and tall T-waves had coronary insufficiency [**11**]. Dressler and Roester considered patients with positive and sharp T-waves to have guarded prognosis [**12**].

**Case IV:**
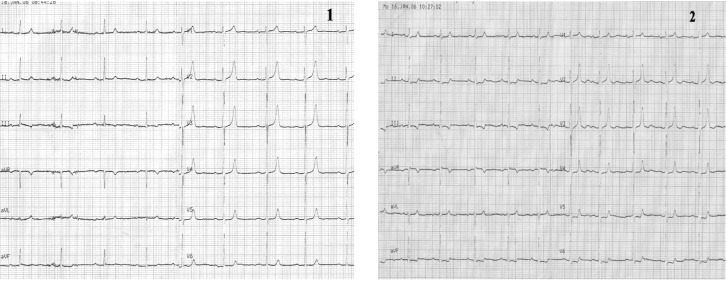
*Electrocardiograms of a 68 year-old woman, with hypertension, diabetes mellitus and dyslipidemia is hospitalized for angina pectoris provoked by rapidly increasing exertion and rest*. **The resting ECG (1)**: sinus rhythm of 60beats/min, AQRS +50°, diphasic T-wave in leads III, aVF and findings of early repolarization in precordial leads with a tall and peaked T-wave. **During episode of angina pectoris** 0.5 mm ST segment depression in leads II, III, aVF and V5-V6, with marked tall, peaked, positive and symmetric T-waves in precordial leads. **Coronarographic:** two-vessel coronary artery disease: stenosis in left anterior descending coronary artery II-III of 70-80% and in right coronary artery of 95%.

## T-wave pseudonormalization

T-wave pseudonormalization – paradox transitory regulation (or positive transformation) of negative T-wave can appear in patients with ischemic cardiopathy.

In patients with resting T-wave inversion, pseudonormalization was slightly more sensitive but less specific than a positive exercise test for the prediction of significant new wall motion abnormalities or decreases in the ejection fraction with exercise.

Although pseudonormalization is not extremely useful alone, the presence or absence of this finding can increase the diagnostic accuracy of exercise electrocardiography in patients with resting T-wave inversion and suspected ischemic heart disease. Thus, when negative T-waves become normal, this change could suggest a worsening of coronary heart disease

**Case V:**
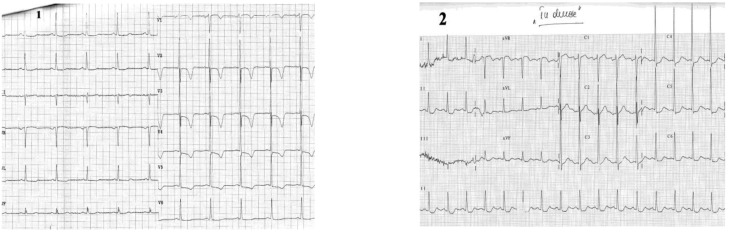
*Electrocardiograms of a 72 year-old man, smoker, with hypertension, dyslipidemia, diabetes mellitus, and angina pectoris during exertion is admitted with worsening of anginal pain within the last 3 months*. **The ECG ** (1): sinus rhythm of 62 beats/min, AQRS +10°, signs of left ventricular hypertrophy, ST segment depression (max. 1.5mm) in leads V2-V6, negative T- wave in leads V1-V5. **During chest pain **(2) note: ST segment depression in leads I, II, aVF and V4-V6, ST segment elevation in lead aVR with positive T-waves in precordial leads. **Coronarographic:** three-vessel coronary artery disease: short stenosis in left anterior descending coronary artery I-II of 90%, long stenosis in left circumflex coronary artery of 90%, and occlusion in right coronary artery from origin.

**Case VI:**
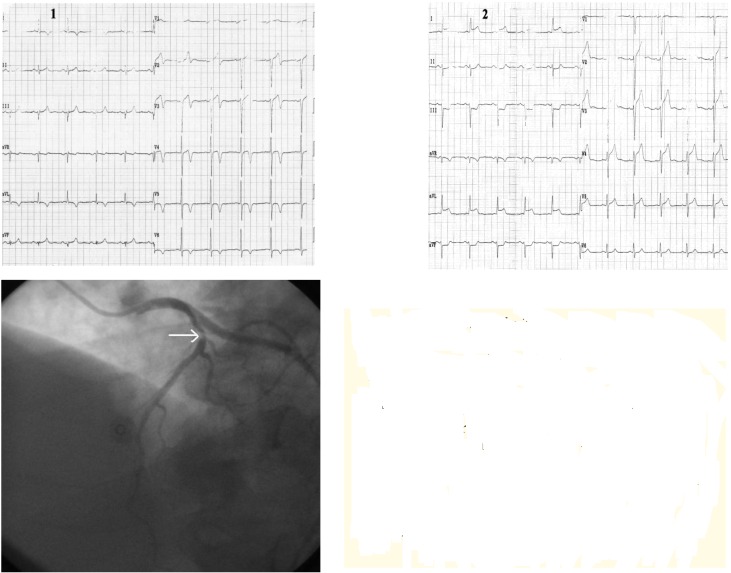
*Electrocardiograms of the 72 year-old hypertensive man admitted with angina during ordinary physical activity in the last month*. **The ECG at admisson (1):** sinus rhythm of 63 beats/ min, AQRS -5°, negative T-wave in leads I, aVL and electrocardiographic signs of left ventricular hypertrophy with mixed abnormalities of repolarization (ST segment elevation in leads V1-V4, diphasic T-waves in leads V2-V4 and negative T-waves in leads V5-V6); **During angina **(2) note: ST segment elevation in leads I, aVL and V2-V5, ST segment depression in II, III, aVF, and positive T-wave in leads I, aVL, V2-V6 **Coronarographic:** one-vessel coronary disease: stenosis in proximal LAD of 80-90%.

## ST – segment elevation

The elevation of the precordial ST segment on the surface electrocardiogram in myocardial ischemia, first noted by Pardee in 1920, has been accepted for many years to be the major diagnostic criteria of acute myocardial infarction or its extention. The electrophysiological correlation was established by Samson and Scher who showed that cute epicardial ST –segment shifts in ischemia were due to accelerated repolarization when surface and intracellular cardiac potentials were recorded simultaneously. The data obtained in these studies suggest the need for caution in the use of acute ST-segment elevation as a predictive index of the extent or severity of myocardial ischemic damage.

There are some characteristics of ST segment elevation in ischemic heart disease, like: persistent ST elevation after acute MI suggests ventricular aneurysm; ST elevation may also be seen as a manifestation of Prinzmetal's (variant) angina (coronary artery spasm); ST elevation during exercise testing suggests extremely tight coronary artery stenosis or spasm (transmural ischemia).

The differential diagnosis of ST elevation implies: a normal variant known as "Early Repolarization" (ST elevation in V2-6 is usually concaved upwards, ending with symmetrical, large, upright T-waves) and acute pericarditis (upwards concaved ST elevation in most leads except for aVR; no reciprocal ST segment depression (except for aVR); unlike "early repolarization", T-waves usually present a low amplitude, and heart rate is usually increased).

**Case VII:**
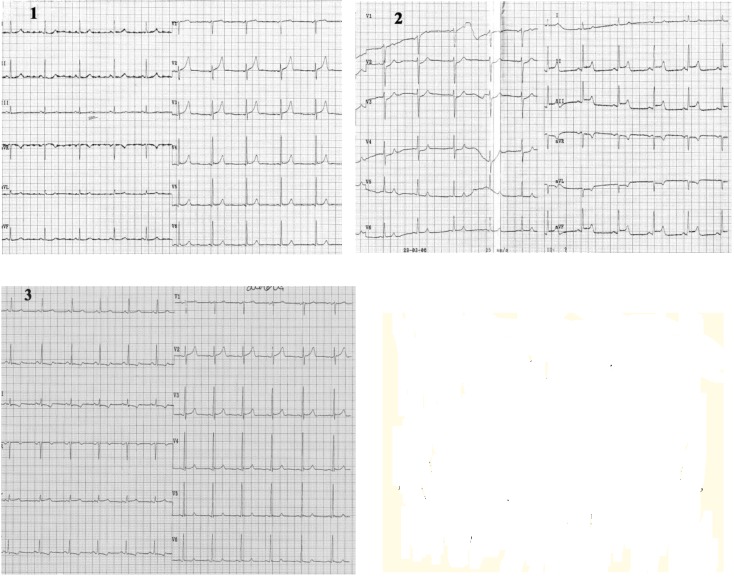
*Electrocardiograms of a 62 year-old man, smoker, with hypertension, dyslipidemia, prior coronary angioplasty with a stent in right coronary artery. He is admitted with a new onset of severe angina within the last month*. **The resting ECG at the admisson** (1): sinus rhythm of 63beats/min, AQRS +50°, without ST-T abnormalities. **During angina **(2) note: ST segment elevation in leads II, III, aVF, and ST depression in leads aVL, V2-V4; at the end of chest pain (3) there are diphasic T-waves in leads II, III and aVF. **Coronarographic:** no significant coronary artery lesions.

**Case VIII:**
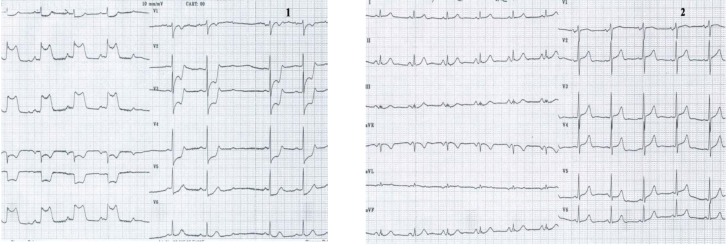
*Electrocardiograms of a 75 year-old man, smoker, with hypertension and stable angina pectoris for 10 years. He is admitted with angina pectoris during rest within the last 3 months*. **The resting ECG at the admisson** (1): sinus rhythm of 68beats/min, marked (7mm) ST segment elevation in leads II, III, aVF, with positive T-wave in leads II, III, aVF, and ST segment depression in leads I, aVR, aVL, V2-V5 At the end of **anginal pain **note (2): decreasing in repolarization abnormalities. **Coronarographic:** three-vessel coronary artery disease.

**Case IX:**
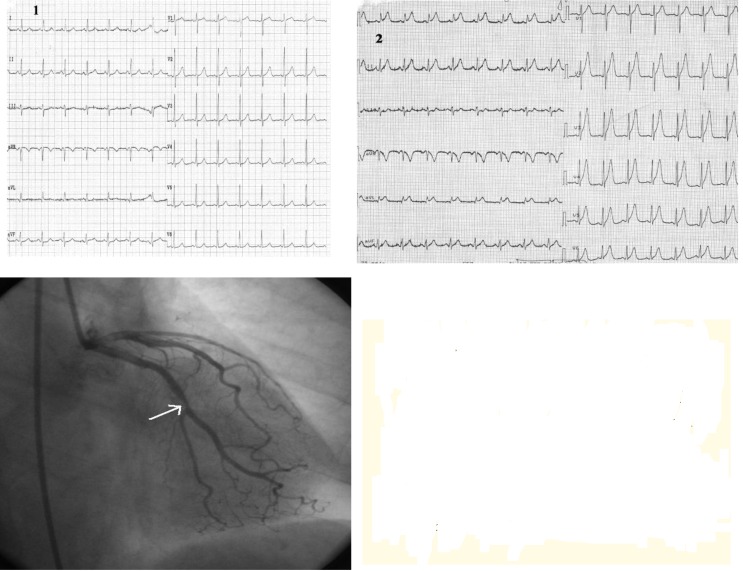
*Electrocardiograms of a 57 year-old woman, smoker, with hypertension, and on imunosupression therapy for cancer of the womb. She is admitted with 2 episodes of angina pectoris after chemotherapy procedure*. **The ECG, without pain ** (1): sinus rhythm of 92beats/min, AQRS +10°, without any repolarization abnormalities. **During chest pain **(2): ST segment elevation in leads I, aVL, V1-V6 and occurrence of a tall, peaked, positive T-wave in precordial leads. **Coronarographic:** one-vessel coronary disease: stenosis in left circumflex coronary artery of 60-70%, at the origin of the first marginal (Mg1) (arrow).

## Negative U-wave

The pathogenesis of U-wave inversion and its clinical value are still not clear although the U-wave was described by Einthoven together with the other electrocardiographic (ECG) waves. The discovery of M cells and their electrophysiology established the cellular basis for repolarization and contributed to our knowledge of U-wave genesis. A negative U-wave is considered a pathologic sign, mostly of cardiac origin, however, with rare exceptions. Hemodynamic changes during diastole in acute ischemia also furnish interesting elements for the interpretation of U-wave changes, and some experimental and clinical studies, besides designating stretch as a cause of U-wave changes. These changes have also proved their value in a more accurate bedside diagnosis and prognosis. A negative U-wave may be the only sign of ischemia; it precedes the typical ST-T changes and increases the sensitivity of the stress test. 

It appears when a vast myocardial territory is involved; it helps locate the culprit vessel,the possible site of myocardial involvement and the expression of extensive ischemia or stunning.

When U-wave changes are the first and only sign of ischemia, they may contribute to a decision regarding the hospital admission of a patient without typical ischemic symptoms. Furthermore, U-wave changes during exercise tests increase their sensitivity [**13**].

Thus, the findings in literature indicate a higher prevalence of U-wave inversion during ischemia than usually reported. They also confirm the correlation between U-wave inversion and extensive myocardial involvement and support the hypothesis that a negative U-wave may be a sign of altered ventricular compliance. It is important to know that a negative U-wave is not always easily detectable and thus, clinicians should look carefully for it, as its presence increases the diagnostic power of electrocardiography.

In literature, there are data which sustain the fact that resting U-wave inversion is a marker of stenosis of the left anterior descending coronary artery [**14**].

**Case X:**
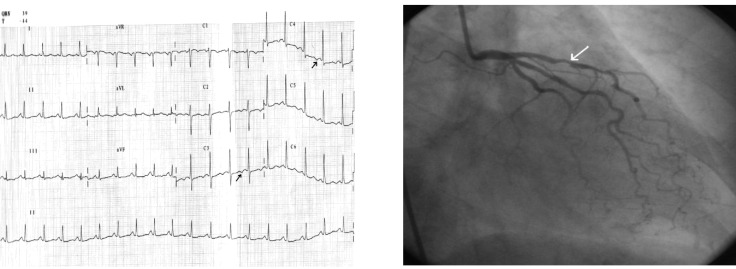
*Electrocardiogram of a 68 year-old woman, with hypertension, dyslipidemia and stable angina pectoris for the last 3 years. She was admitted in hospital with typical angina with marked limitation of ordinary activities over the last month*. **The ECG (with pain)**: sinus rhythm of 130 beats/min, AQRS +45°, diffuse flat T-wave, and negative U- wave in leads V2-V6. **Coronarographic:** one-vessel coronary artery disease: stenosis in the proximal left anterior descending coronary artery of 75% (arrow).

## The prolonged QTc interval

The QTc interval prolongs in 100% of the patients with early transmural ischemia. When compared with clinically accepted indexes of transmural ischemia it represents the earliest ECG abnormality [**15**].

**Case XI:**
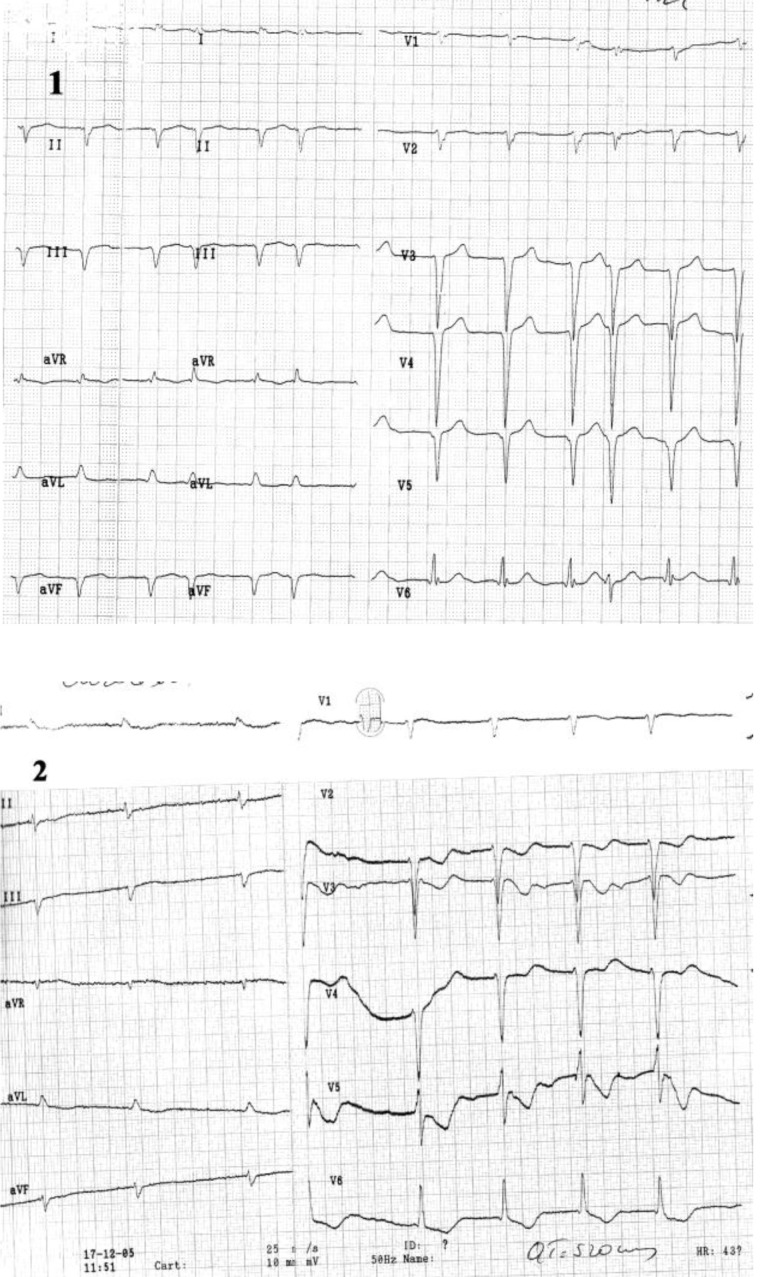
*Electrocardiograms of a 69 year-old man, with dilated cardiopathy, is admitted with chest pain and dyspnea during exertion*. **The ECG (patient without pain)**: atrial fibrillation of 100 beats/min, QS complex in leads II, III, aVF, and V2-V5 (1). During pain: ST segment depression in leads I, aVL, V2-V6, prolonged QTc interval (QTc = 520msec) and diphasic T-wave in leads V2-V6 (2). **Coronarographic:** three-vessel coronary artery disease (stenosis in LADII of 80%, stenosis in Mg 1 of 70% and occlusion in right coronary artery).

**Case XII:**
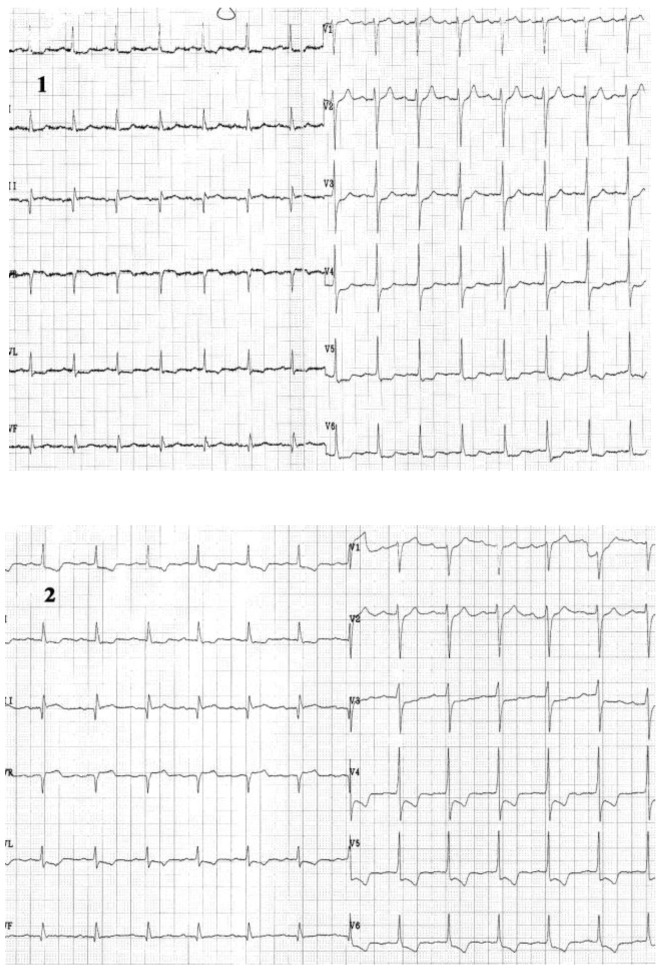
*Electrocardiograms of a 75 years old man, corpulent, with dyslipidemia, hypertension, diabetes mellitus, prior myocardial infarction, is admitted with angina pectoris at rest*. The **ECG without pain ** (1): sinus rhythm of 93 beats/min, AQRS +10°, ST segment depression in leads I, aVL, V3-V6. During pain (2): increased ST segment depression and occurrence of negative T-wave in I, aVL, V4-V6, and ST segment elevation in III, aVR. **Coronarographic:** three-vessel coronary disease (stenosis in LAD II of 90%, occlusion in Cx, hypoplasia of right coronary artery.

In conclusion, the electrocardiogram is a very useful non-invasive cardiac investigation in the assessment of the myocardial ischemia. We selected these cases in order to exemplify some electrocardiographic chances and their correlation with the degree of coronary heart disease.

**Figure:**
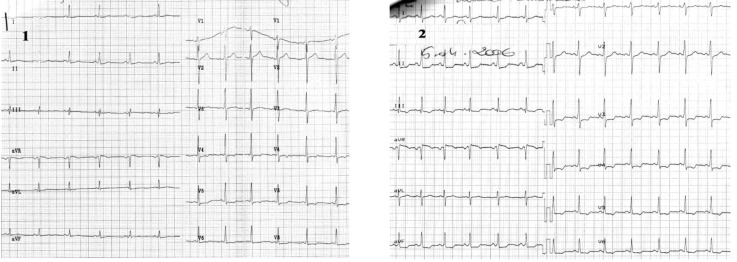
*Electrocardiograms of a 38 year-old female, without a previous cardiac disease, is admitted with severe chest pain new onset within the last 3 weeks*. The **ECG without pain ** (1): sinus rhythm of 92 beats/min, AQRS +30°, minor ST- segment depression in leads I, II, V4-V6. **During chest pain ** (2): ST segment depression in leads III, aVF, V3 , increased ST-segment depression in leads II, V4-V6, with ST-segment elevation in aVR . **Coronarographic:** no significant coronary artery lesions.
